# Treatise on Therapeutics

**Published:** 1880-10

**Authors:** 


### BIBLIOGRAPHICAL.

TREATISE ON THERAPEUTICS. Translated by D. F. Lincoln, M.D. From the French of A. Trosseau, Professor of Therapeutics in Faculty Med., Paris, etc., etc., and H. Pidoux, Member of the Academy of Medicine, etc., etc. Ninth edition. Revised and enlarged, with the assistance of Constantine Paul, Professor Agrege in the Faculty of Medicine, Paris, etc., etc. Vol. II. Wm. Wood & Co., New York. 1880.

Armand Trosseau bears a world-wide reputation as a medical reformer, and few there are who are not acquainted, in some detail, with his doctrines. In the history of medicine he may be ranked with Galen and Hunter. As a shrewd and practical observer he is not inferior to Dupuytren or Rush, and has contributed much to the progress of medicine as an art and science. The incorporation of his doctrines and observations in their series of "Standard Medical Authors," by Messrs. Wm. Wood & Co., is an enterprise calculated to be profitable, as well as instructive to all medical readers, and will, no doubt, meet with a worthy appreciation. We are unable, at present, to render a detailed review of the work, but feel assured that its merits will not go unrequited, or unrecognized.

We have received, through the efficient agent of Messrs. Wood & Co., Mr. Dalston, of this place, the two volumes comprising this work, and are glad we are enabled to make so valuable an edition to our library.

